# 5-HT_2_ receptors mediate functional modulation of GABAa receptors and inhibitory synaptic transmissions in human iPS-derived neurons

**DOI:** 10.1038/srep20033

**Published:** 2016-02-03

**Authors:** Haitao Wang, Lingli Hu, Chunhua Liu, Zhenghui Su, Lihui Wang, Guangjin Pan, Yiping Guo, Jufang He

**Affiliations:** 1 Key Laboratory of Regenerative Biology, South China Institute for Stem Cell Biology and Regenerative Medicine, Guangzhou Institutes of Biomedicine and Health, Chinese Academy of Sciences, Guangzhou, 510530, China; 2 Guangdong Provincial Key Laboratory of Stem Cell and Regenerative Medicine, South China Institute for Stem Cell Biology and Regenerative Medicine, Guangzhou Institutes of Biomedicine and Health, Chinese Academy of Sciences, Guangzhou, 510530, China; 3 Department of Pathology, Dalian Medical University, Dalian, 116044, China; 4 Department of Biomedical Sciences, City University of Hong Kong, Tat Chee Avenue, Kowloon, Hong Kong SAR, China

## Abstract

Neural progenitors differentiated from induced pluripotent stem cells (iPS) hold potentials for treating neurological diseases. Serotonin has potent effects on neuronal functions through multiple receptors, underlying a variety of neural disorders. Glutamate and GABA receptors have been proven functional in neurons differentiated from iPS, however, little is known about 5-HT receptor-mediated modulation in such neuronal networks. In the present study, human iPS were differentiated into cells possessing featured physiological properties of cortical neurons. Whole-cell patch-clamp recording was used to examine the involvement of 5-HT_2_ receptors in functional modulation of GABAergic synaptic transmission. We found that serotonin and DOI (a selective agonist of 5-HT_2A/C_ receptor) reversibly reduced GABA-activated currents, and this 5-HT_2A/C_ receptor mediated inhibition required G protein, PLC, PKC, and Ca^2+^ signaling. Serotonin increased the frequency of miniature inhibitory postsynaptic currents (mIPSCs), which could be mimicked by α-methylserotonin, a 5-HT_2_ receptor agonist. In contrast, DOI reduced both frequency and amplitude of mIPSCs. These findings suggested that in iPS-derived human neurons serotonin postsynaptically reduced GABAa receptor function through 5-HT_2A/C_ receptors, but presynaptically other 5-HT_2_ receptors counteracted the action of 5-HT_2A/C_ receptors. Functional expression of serotonin receptors in human iPS-derived neurons provides a pre-requisite for their normal behaviors after grafting.

Induced neurons from neural stem cells of embryonic stem cell (ES) and induced pluripotent stem cells (iPS) origins not only exhibited neuronal morphology with extensive axon and dendrites, but also possessed mature electrophysiological properties such as repetitive action potentials in response to current stimulation and integrated synaptic connections with host both *in vitro* and *in vivo*^1^,^2^,^3^. The iPS-derived neurons recapitulated symptoms of patients with neurological diseases at cellular and synaptic levels, providing a rather distinct insight into the underlying mechanisms^4^,^5^. Autologous neuronal progenitors obtained from iPS would provide enough source cells for transplantation, which hold great potentials to treat neurological disorders such as Parkinson’s disease, schizophrenia and stroke^6^,^7^,^8^,^9^,^10^,^11^,^12^,^13^. Input-specific long-term potentiation^14^, homeostatic plasticity, as well as BDNF induced synaptic changes^15^ were reported for ES-derived neurons, further physiologically supporting the functional integrity of transplanted progenitors.

The development and function of the cerebral cortex is subjected to massive monoaminegic modulation. Serotonin as one of important neuromodulators participates in many neural processes including synaptic transmission, mood, learning and memory^16^,^17^,^18^,^19^. Serotonergic fibers from raphe nucleus diffusely projecting to the brain exert its modulatory effect through multiple receptor subtypes (1–7)^19^. So far amounting evidences revealed the existence of glutamate and GABA receptors in neural stem cell derived neurons^1^,^2^ and even the role of neuromodulators in the proliferation and differentiation of neural stem cells^20^,^21^; however few studies addressed the neuromodulation mediated by serotonin and its receptors. The transplanted neurons have to communicate with host neurons and to be modulated normally to achieve satisfactory therapeutic efficacy. Activation of 5-HT_2_ receptors has been reported to broadly regulate receptor function and neuronal excitability^7^,^22^,^23^,^24^,^25^,^26^. Functional expression of serotonin receptors in human iPS-derived neurons would endow them with the ability to receive normal serotonergic modulation from the host after grafting, and to serve as a cell model to screen serotonin related neuropsychological drugs. Here we differentiated iPS into forebrain-like cortical neurons, and whole-cell patch-clamp recording technique was adopted to investigate the involvement of 5-HT_2_ receptors in the modulation of GABAa receptor function and inhibitory synaptic transmissions.

## Results

### Acquisition of forebrain-like neural progenitor cells from human iPS

**Figure 1:**
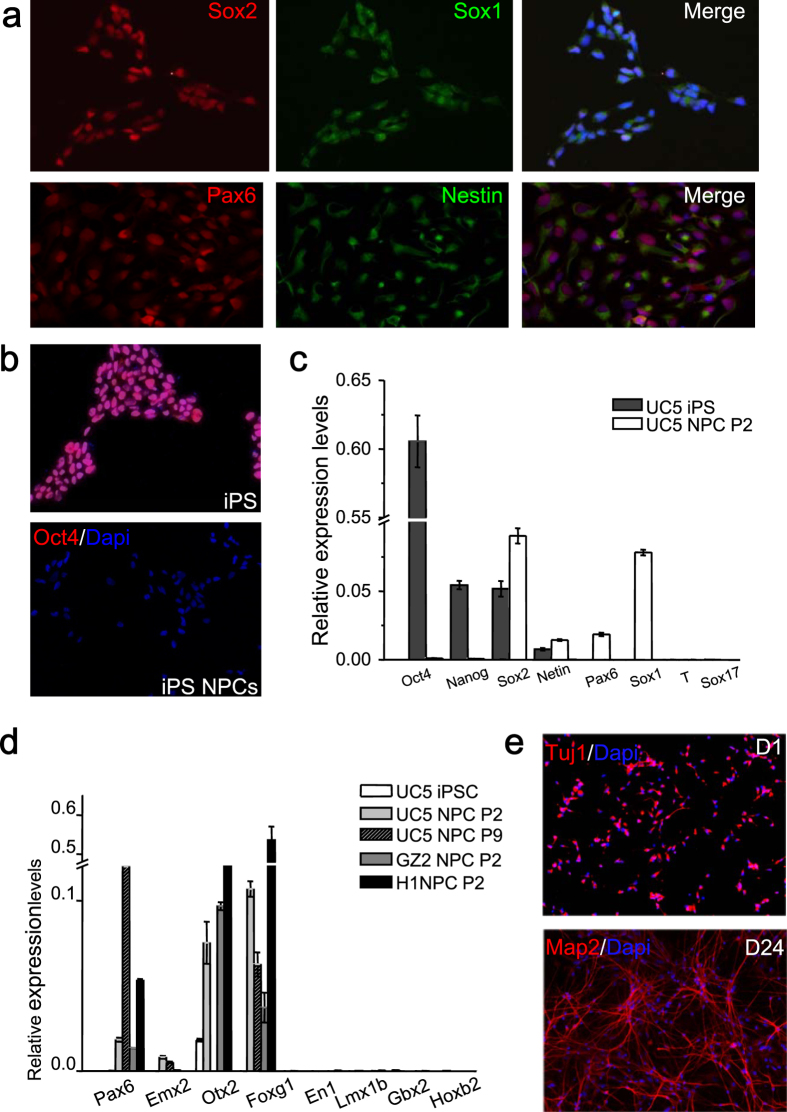
Acquisition and identification of forebrain-like neurons from iPS. (**a**), Immunostaining showed iPS-derived NPCs expressing Pax6, Sox1 and Nestin of high percentages while no expression of pluripotent marker Oct4 (**b**), which was further confirmed by Q-PCR, (**c**), however, the mesoderm marker (T) and endoderm marker (Sox17) were undetectable in these NPCs. (**d**), Q-PCR also confirmed the dorsal forebrain identity of the NPCs, which remained the same characteristics even after long-term passages. Pax6 and Emx2 (not done in H1 NPC): markers for dorsal CNS (central nervous system), Otx2 (not done in UC5 NPC P9) and Foxg1: forebrain markers, En1 and Lmx1b: midbrain markers, Gbx2 and Hoxb2: hindbrain markers. P2, Passage 2; P9, Passage 9. (**e**), Immunoflurescent photographs showed the NPCs were highly neurogenic with high expression of typical neuronal markers (early Tuj1 and mature Map2). D1: 1 day post-differentiation; D24: 24 day post-differentiation.

Neural progenitor cells (NPCs) were derived from human iPS reprogrammed from urine cells (UC5) or fibroblasts (GZ2) as described before^27^,^28^, as well as ES (H1) as a control. Unless otherwise specified, the majority of the results shown here were obtained from iPS line UC5, which was derived by using a feeder-free, serum-free and virus-free method without oncogene c-MYC, representing a more promising translational application for regenerative medicine. These NPCs expressed Sox1, Pax6 and Nestin of high percentages (Fig. 1a), without mesodermal and endodermal contamination (undetectable markers T and Sox17 by Q-PCR), whereas pluripotency markers including Oct4 and Nanog stopped to express (Fig. 1b,c). The NPCs can stably expand in both suspension and adhesion conditions in neural expanding medium containing EGF and bFGF. More importantly, these cells possessed dorsal forebrain regional identity, which maintained even after long-term passages (Fig. 1d). The NPCs were highly neurogenic, and showed typical neuronal morphology and gene expression profile upon differentiation and maturation (Fig. 1e).

### Basic electrophysiological properties of iPS-derived forebrain neurons

**Figure 2:**
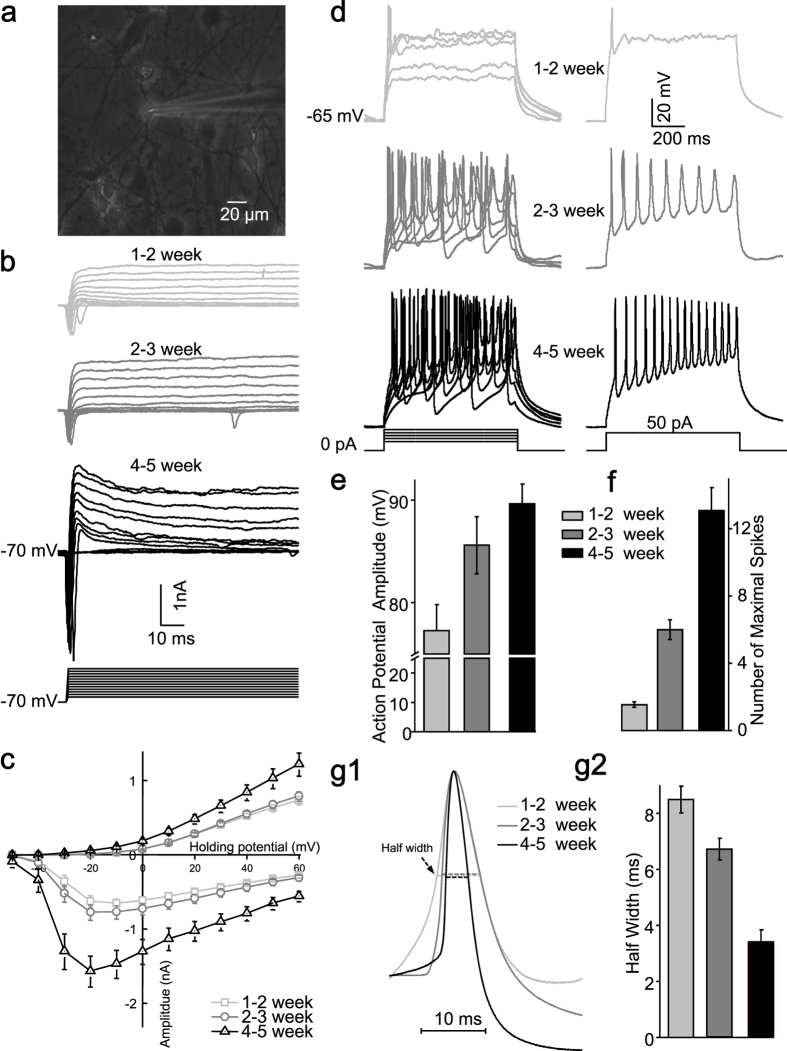
Intrinsic membrane properties of iPS-derived neurons. (**a**), Phase contrast micrograph showing a typical iPS-derived neuron being approached by a patching pipette. (**b**), Representative voltage-gated ion currents recorded from an iPS-derived neuron at three developmental stages (upper panel: 1-2 week; middle panel: 2-3 week; lower panel: 4-5 week). The whole cell Na^+^ (inward) and K^+^ (outward) currents were elicited by test pulses to potentials between −50 mV and + 60 mV in steps of 10 mV from a holding potential of −70 mV (lower panel). (**c**), Quantitative analysis of current-voltage relationship respectively of Na^+^ (negative) and steady sate K^+^ (positive) ion currents at different development stages (1-2 week, n = 50; 2-3 week, n = 50; 4-5 week, n = 31). (**d**), Representative traces of action potentials (left panel) in response to injected current steps (20, 30, 40, 50, 60 pA) with 800 ms duration at three developmental stages, Action potentials (right panel) evoked by 50 pA injected current (lowest panel). Quantitative analysis of action potential amplitude (**e**), number of maximal spikes in response to 800 ms current injection (**f**) and half width (**g**) of the action potential at three developmental stages (1-2 week, n = 18; 2-3 week, n = 18; 4-5 week, n = 13). The waveforms of scaled action potentials revealed gradual narrowing of the half width (g1).

**Figure 3:**
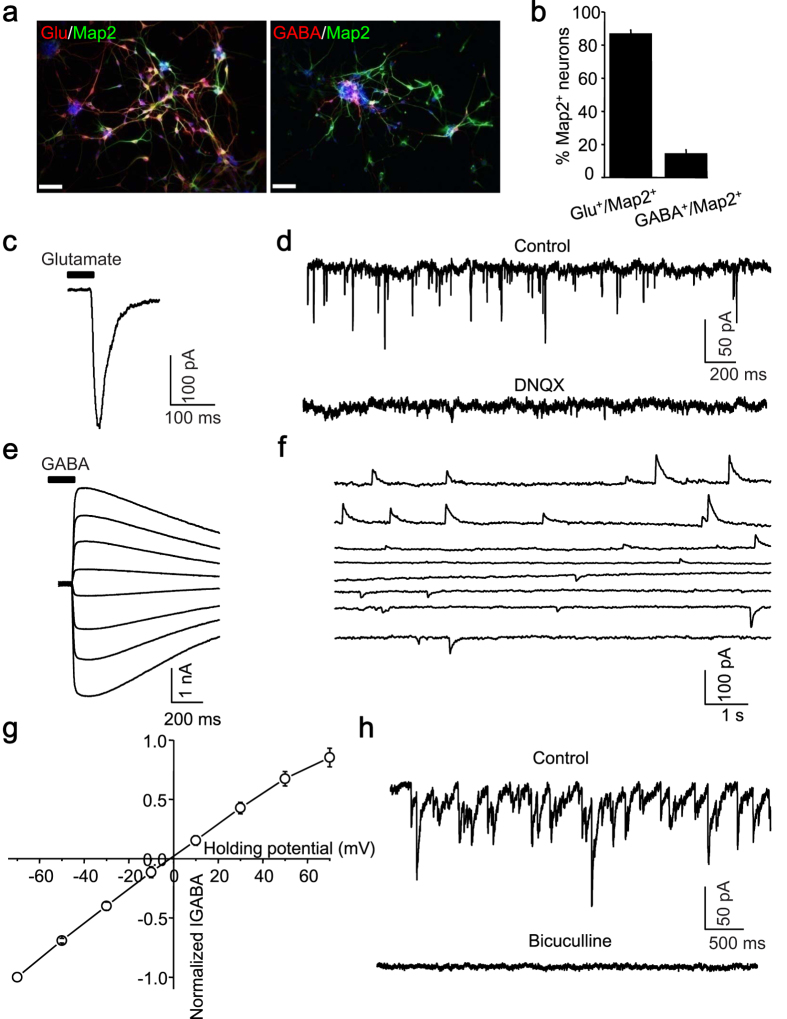
Characterization of neuronal subtypes and functional analyses of receptors currents in iPS-derived neurons. (**a**), The majority of spontaneously differentiated iPS-derived neurons were immunopositive to glutamate (left), with a small portion being GABA^+^ (right). Scale bar: 50 μm. (**b**), Quantitative analysis of glutamate^+^ and GABA^+^ among Map2 positive neurons. (**c**), A current response to glutamate (1 mM) delivered through puffing electrode (15 psi, 30 ms). (**d**), The pharmacologically isolated glutamatergic spontaneous excitatory postsynaptic currents could be completely blocked by the AMPA/KA receptor antagonist (DNQX, 20 μM). The current response to GABA (1 mM) (**e**) delivered through puffing electrode (15 psi, 30 ms) and spontaneous GABAergic inhibitory postsynaptic currents (**f**) starting from a holding potential of –70 mV to 70 mV in a step of 20 mV. (**g**), Current-Voltage curve of GABA evoked response revealing its reversal potential at about 0 mV (n = 4). (**h**), Pharmacologically isolated GABAergic spontaneous inhibitory postsynaptic currents could be completely blocked by a selective GABAa receptor antagonist (Bicuculline, 10 μM).

Next, electrophysiological studies were performed on passage 3–8 NPCs-differentiated neurons (Fig. 2a). The majority of them differentiated into glutamate positive neurons (glutamate^+^, 86.80 ± 0.97%, n = 6; GABA^+^, 14.19 ± 1.07%, n = 6) (Fig. 3a,b). Voltage-gated sodium and potassium channels of these induced neurons experienced the developmental changes (Fig. 2b,c). Only sparse spikes with lower amplitude and longer duration were elicited at early stages; and progressively maturing neurons displayed repetitive action potentials in response to current injections (Fig. 2d–g), which is consistent with previous reports^29^,^30^,^31^.

Moreover, the puffed glutamate (1 mM) evoked an inward current (Fig. 3c) and spontaneous excitatory postsynaptic currents was blocked by DNQX (20 μM), a selective glutamate receptor antagonist, which indicated that glutamate receptors mediated excitatory synaptic transmission (Fig. 3d). The iPS-derived neuron also responded to puffed 1 mM GABA with an inward current (Fig. 3e). The GABAergic spontaneous IPSCs (Fig. 3f) and GABA-activated currents reversed at 0.92 ± 2.33 mV (n = 4, Fig. 3g), which approximated the theoretical equilibrium potentials of Cl^−^ with CsCl-based internal solutions^32^. Bicuculline (10 μM), a selective GABAa receptor antagonist completely blocked GABA-activated current and spontaneous IPSCs (Fig. 3h). These indicated that GABAa receptors on the postsynaptic membrane mediated inhibitory synaptic transmission. These electrophysiological evidences suggested that iPS-derived neurons behaved as normal neurons communicating each other through fundamental synaptic connections. To minimize developmental difference of serotonin receptors, at least 4-week-old mature iPS-derived neurons were used to examine the serotonergic modulation.

### 5-HT_2A/C_ receptor mediated reduction of GABA-activated current

**Figure 4:**
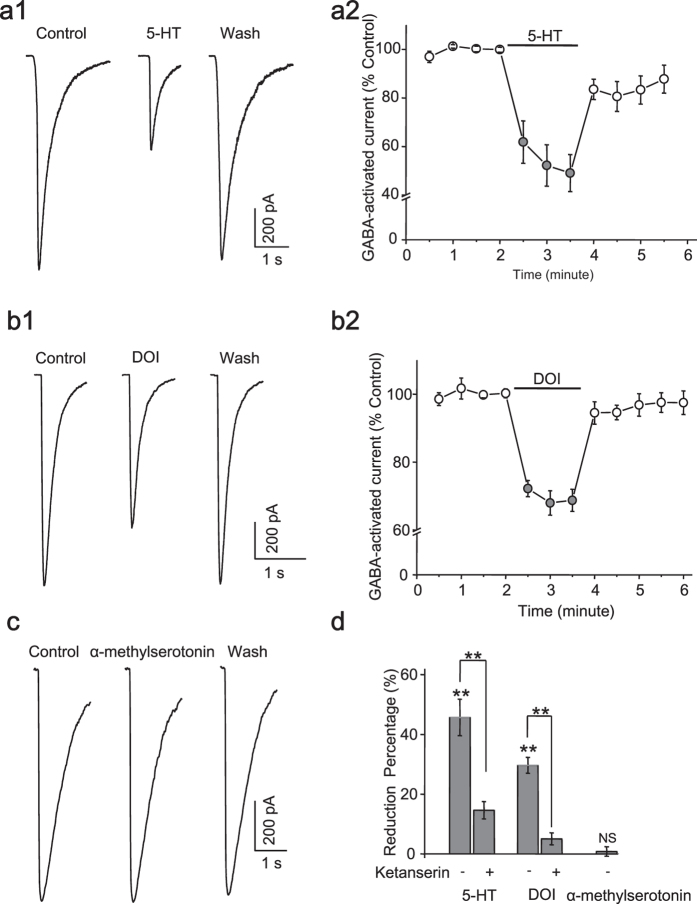
5-HT reduced GABA current mainly through activation of 5-HT_2A/C_ receptors. The representative current traces showing the depressive effect of 5-HT (40 μM) (**a1**) and DOI (20 μM) (b1) on GABA currents, which was recovered after washout. (**a2**) and (**b2**) showed the time course of GABA currents when 5-HT and DOI were applied. (**c**), Representative traces showing no effect of α-methylserotonin (30 μM) on GABA currents. (**d**), Quantification data showing the reduction of GABA currents by 5-HT (n = 5), DOI (n = 10) and α-methylserotonin (n = 6). The deceasing effect was largely attenuated by ketanserin (40 μM, a selective 5-HT_2A/C_ receptor antagonist) (n = 4). **p  <0.01. NS, no significant.

Serotonergic projections diffusely innervate the central nervous system via multiple types of receptors, and most 5-HT receptors are metabotropic receptors that are involved in the regulation of neuronal function. Serotonin at 40 μM significantly and reversibly reduced GABA activated currents (reduction%: 45.68 ± 6.14%, n = 5, *p* = 0.002, Student’s *t*-test) (Fig. 4a,d) in the iPS-derived neurons. Since 5-HT_2_ receptors were reported to responsibly modulate GABAa receptors in cortical neurons, we applied DOI (20 μM), a 5-HT_2A/C_ receptor agonist to examine whether 5-HT_2A/C_ receptors accounted for the reduction. We found that DOI similarly reduced GABA-activated currents as serotonin (reduction%: 29.64 ± 2.65%, n = 10, *p* < 0.001, Student’s *t*-test) (Fig. 4b,d). However α-methylserotonin, the agonist of 5-HT_2_ receptor, did not cause any change of GABA-activated currents (reduction%: 0.80 ± 1.61%, n = 6, *p* = 0.612, Student’s *t*-test) (Fig. 4c,d), suggesting a counteractive interaction between subtypes of 5-HT_2_ receptors. The depressive effect of 5-HT and DOI was also replicated in the induced neurons reprogrammed from another cell line (GZ2) (reduction%: 5-HT: 52.87 ± 9.91%, n = 8, *p* = 0.001; DOI: 28.14 ± 5.37%, n = 6, *p* = 0.001, Student’s *t*-test). The reducing effect of 5-HT and DOI on GABA current could be largely attenuated by ketanserin (40 μM), a selective antagonist of 5-HT_2A/C_ receptor (reduction%: 5-HT: 14.59 ± 2.91%, n = 4; DOI: 5.04 ± 1.99%, n = 4) (5-HT + ketanserin *vs*. 5-HT, *p* < 0.005; DOI + ketanserin *vs*. DOI, *p* < 0.001, Student’s *t*-test) (Fig. 4d), demonstrating that the reduction of GABA-activated current by serotonin could be majorly ascribed to the activation of 5-HT_2A/C_ receptors at the postsynaptic membrane in human iPS-derived neurons. Notably, the larger reduction caused by 5-HT than DOI, as well as the incomplete blocking by ketanserin, implied the contribution of other 5-HT subtype receptors. And we did observe that 8-hydroxy-2-dipropylaminotetralin (DPAT, 20 μM), a selective 5-HT_1A_ receptors agonist, caused a reversible reduction of GABA-activated current (control: 492.72 ± 118.36 pA; DPAT: 435.56 ± 98.13 pA; n = 5, *p* = 0.035, Student’s *t*-test).

### G-protein is required for 5-HT_2A/C_ receptor mediated reduction of GABA-activated currents

**Figure 5:**
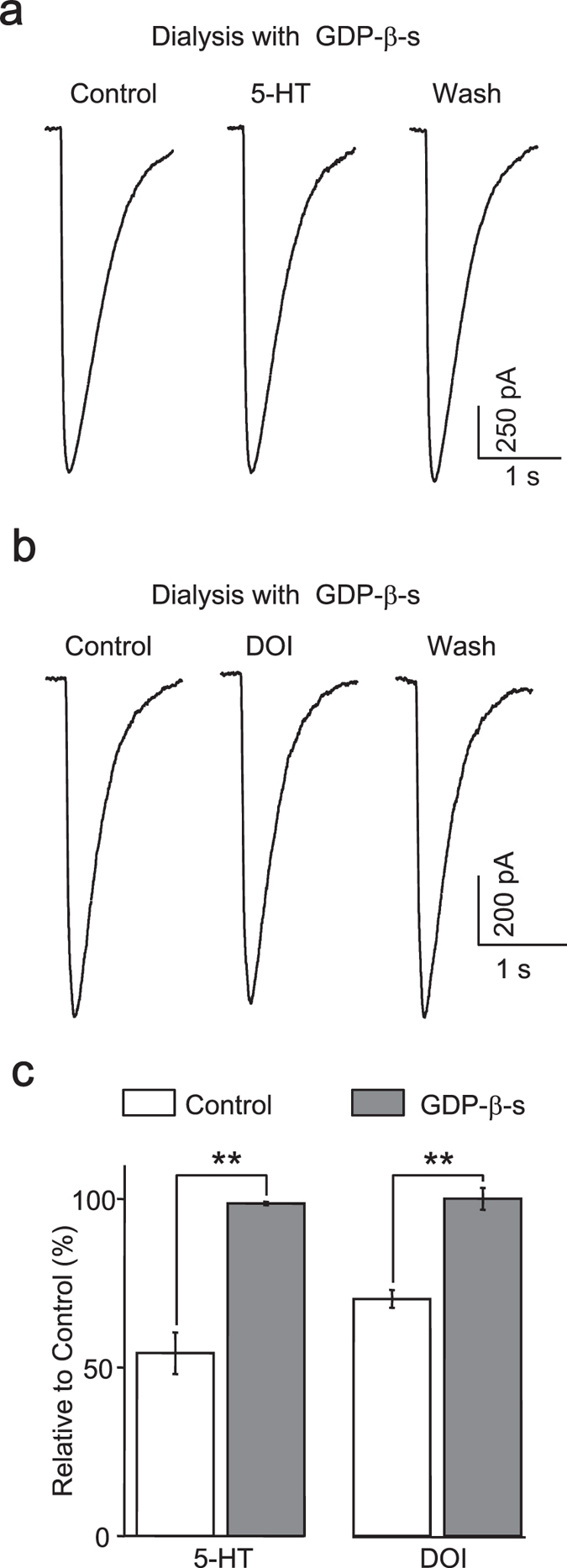
G-protein is required for 5-HT_2A/C_ receptor mediated reduction of GABA-activated currents. In the presence of GDP-b-s (500 μM), which was included in the pipette solution, 5-HT (**a**) and DOI (**b**) failed to attenuate the GABA currents. (**c**), Quantification data showing no effect of 5-HT (n = 6) and DOI (n = 6) on GABA current after blocking the activation of G-protein.

Activation of G-protein is the prerequisite of the action of G-protein-coupled receptors (GPCR), and all 5-HT receptors except ionic 5-HT_3_ were coupled to different G proteins^33^. We then examined whether activation of G-protein was the prerequisite of the action of 5-HT and DOI on GABA current. When dialysis of GDP-β-s (0.5 mM) constitutively prevented G-protein complex from dissociating into active subunits of G_α_ and G_βγ,_ the depressive effect of 5-HT (Fig. 5a) and DOI (Fig. 5b) on GABA activated currents was completely blocked (5-HT: 98.68 ± 0.49% of control, n = 6; DOI: 100.10 ± 3.21% control, n = 6) (5-HT + GDP-β-s *vs*. 5-HT, *p* < 0.005; DOI + GDP-β-s *vs*. DOI, *p* < 0.001, Student’s *t*-test) (Fig. 5c). These data indicated that negative regulation of GABAa receptors by 5-HT_2_ receptors was dependent on the activation of G-protein. In this and following recordings of applying intracellular blockers, it must be mentioned that at least 5–10 minutes’ waiting period was needed to achieve a successful inhibition^34^.

### Calcium signaling is involved in the modulation of GABA current by 5-HT_2A/C_ receptor

**Figure 6:**
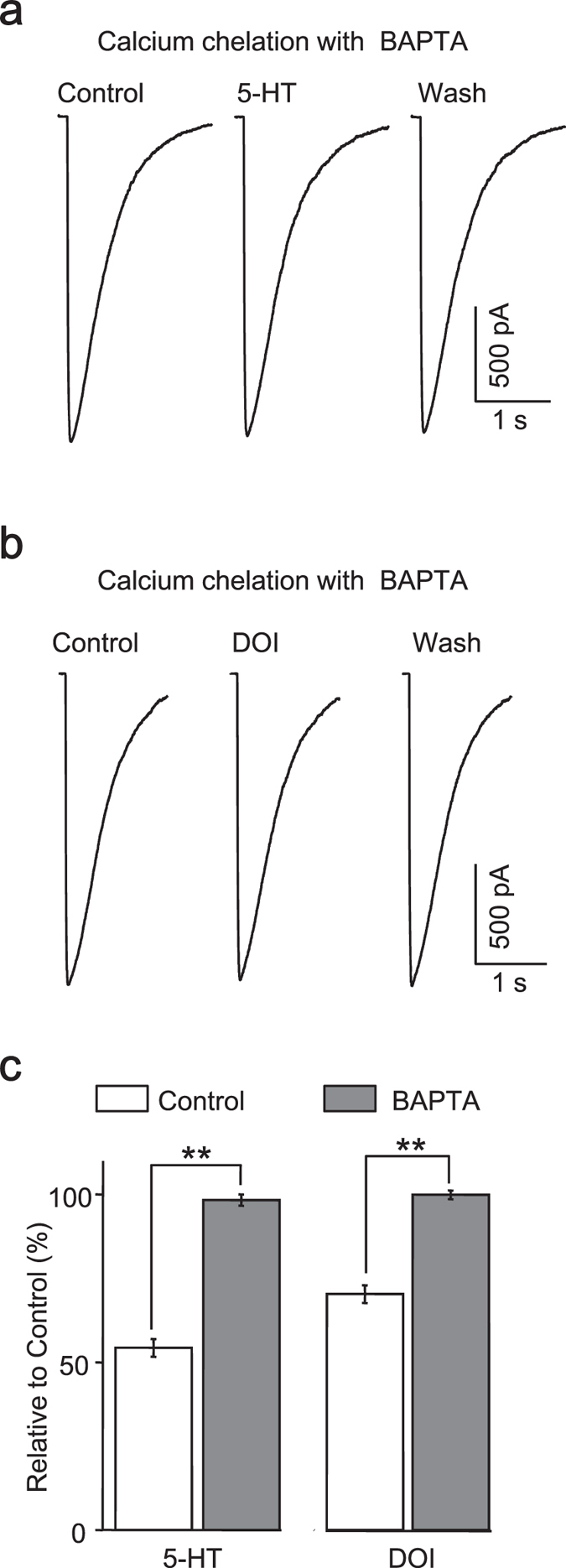
Calcium signaling is involved in the modulation by 5-HT2A/C receptor. Inclusion of BAPTA (10 mM) in the pipette solution prevented the modulatory effect of 5-HT (**a**) and DOI (**b**) on GABA currents. (**c**), Quantification data showing that intracellular calcium signaling was required to cause the reduction of GABA currents by 5-HT (n = 7) and DOI (n = 5).

Ca^2+^ as an important second messenger participates in many physiological processes, and its rise triggered by IP3 would have a potential impact on GABAa receptors as reported before. BAPTA as a potential and rapid chelator of intracellular Ca^2+^ was used to test the involvement of calcium signaling in the process of 5-HT_2A/C_ receptor mediated reduction of GABA current. When BAPTA (10 mM) was included in the pipette solutions, interestingly both 5-HT (Fig. 6a) and DOI (Fig. 6b) lost their ability to change the amplitude of GABA currents (5-HT: 98.39 ± 1.66%, n = 7; DOI: 99.94 ± 1.32%, n = 5) (5-HT + BAPTA *vs*. 5-HT, *p* < 0.005; DOI + BAPTA *vs*. DOI, *p* < 0.001, Student’s *t*-test) (Fig. 6c).

### Phospholipase C and protein kinase C act as downstream signaling of 5-HT_2A/C_ receptors

**Figure 7:**
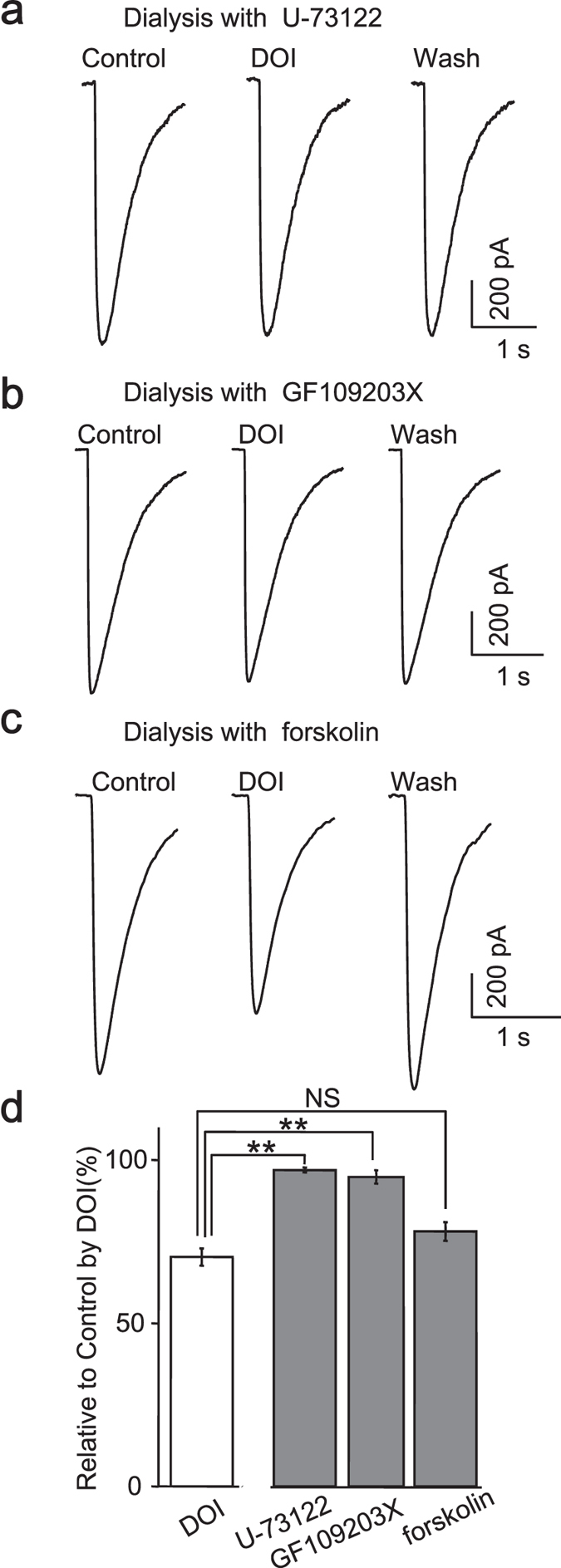
Phospholipase C and protein kinase C act as downstream signaling of 5-HT_2A/C_ receptors. Inclusion of U-73122 (5 μM, n = 6, (**a**) GF 109203X (2.5 μM, n = 5, (**b**) in the pipette solution prevented the modulatory effect of DOI on GABA currents, but forskolin (10 μM, n = 5, c) failed to achieve the occluding effect. (**c**), Quantification data showing that activation of PLC-PKC pathway caused the reduction of GABA currents.

Gq protein-coupled 5-HT_2A/C_ receptor theoretically would promote phospholipase C (PLC) to hydrolyze phosphoinositol lipids on the membrane into ionsitol-1,4,5-triphosphate (IP3) and diacylglycerol (DAG), and then the rise of Ca^2+^ and activation of protein kinesis C (PKC) would change the phosphorylation of GABAa receptors. We examined whether the same mechanism occurred in iPS-derived neurons by intracellularly delivering correspondent inhibitors. As expected, when PLC activity was blocked with U-73122 (5 μM) in recording pipette, GABA- activated currents remained unchanged in the presence of DOI (97.03 ± 0.73% control, n = 6) (DOI + U-73122 *vs*. DOI, *p* < 0.001, Student’s t-test) (Fig. 7a,d), supporting the involvement of PLC in 5-HT_2A/C_ receptors mediated regulation of GABAa receptors.

Similar to those reported in rat cortical neurons, the inclusion of GF109203X (2.5 μM), a PKC inhibitor, significantly abolished the reduction of GABA currents by DOI (94.85 ± 2.05% control, n = 5) (DOI + GF109203X *vs*. DOI, *p* < 0.001, Student’s t-test) (Fig. 7b,d), implying that the kinase activity of PKC was indispensable for the downstream signaling following activation of 5-HT_2_ receptor. This conclusion was further confirmed by the failure of adenylyl cyclase activator (forskolin, 10 μM) to occlude the effect of DOI (78.16 ± 2.86% of control, n = 5) (DOI + forskolin *vs*. DOI, *p* = 0.063, Student’s *t*-test) (Fig. 7c,d).

Taken together, these data suggested that PLC and PKC act as downstream signaling cascade to achieve the modulation of GABAa receptors by 5-HT_2A/C_ receptors in human iPS-derived forebrain neurons.

### 5-HT_2_ receptors mediated modulation of spontaneous inhibitory postsynaptic currents

**Figure 8:**
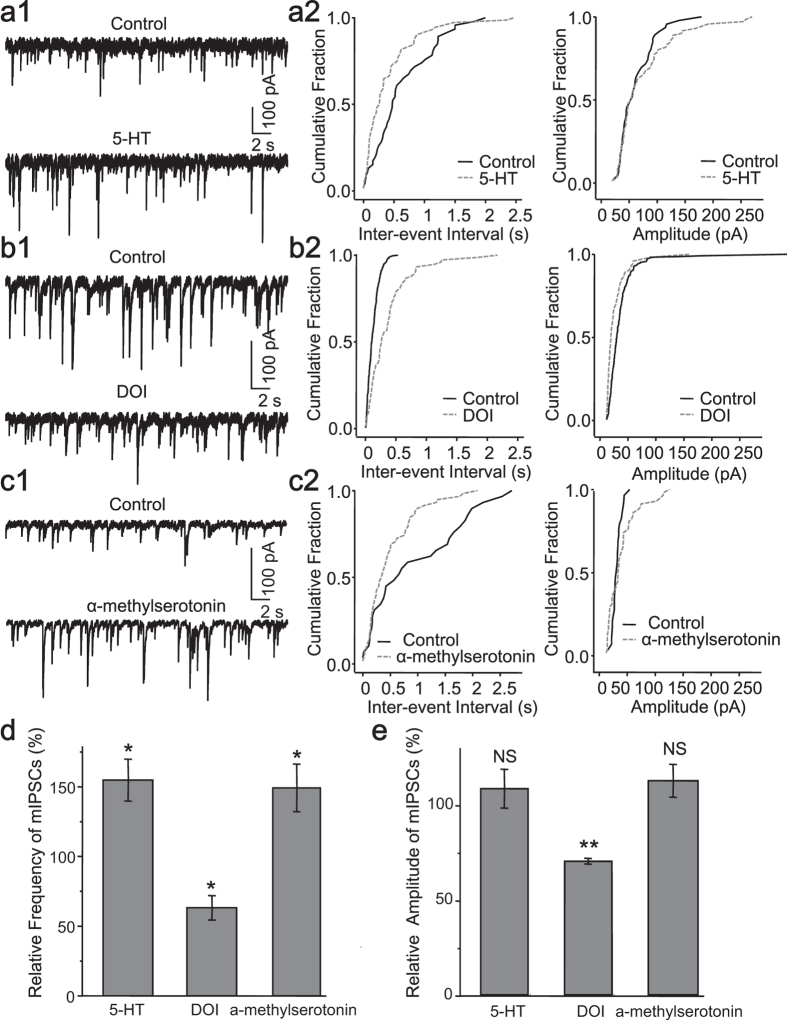
5-HT_2_ receptors mediated modulation of spontaneous inhibitory postsynaptic currents. The representative traces showing the mIPSCs (**a1, b1, c1**), and the cumulative plots of the mIPSCs (frequency: left panel; amplitude: right panel) in the absence and presence of the 5-HT (**a2**), DOI (**b2**) and α–methylserotonin (**c2**). (**d**), Quantification data showing the effect of the agonists of 5-HT_2_ receptors on the frequency of mIPSCs. (**e**), Quantification data showing the effect of the agonists of 5-HT_2_ receptors on the amplitude of mIPSCs (5-HT, n = 5; DOI, n = 5; α-methylserotonin, n = 6).

GABA delivered through pressure puffing directly activated GABA receptors on the cell surface, thus 5-HT receptors on postsynaptic membrane contributed to the above mentioned modulation. Notably, serotonin also exerts a powerful influence on the presynaptic neurotransmitter release^32^. In order to understand the contribution from presynaptic 5-HT receptors, we performed miniature IPSCs (mIPSCs) recording in iPS-derived neuronal networks in the presence of 1 μM TTX to block the spontaneous action potentials. In contrast to the depressive modulation at postsynaptic site, serotonin robustly enhanced the frequency, but not the amplitude of mIPSCs (frequency: 154 ± 15% of control, *p* = 0.022; amplitude:108 ± 10% of control, *p* = 0.453, Student’s *t*-test, n = 5, Fig. 8a1,d,e), and this could be mimicked by agonist of 5-HT_2_ receptor, α-methylserotonin (frequency: 149 ± 17% of control, *p* = 0.034; amplitude:112 ± 8% of control , *p* = 0.200, Student’s *t*-test, n = 6, Fig. 8c1,d,e). But DOI still caused a reduction of both frequency and amplitude of mIPSCs (frequency: 63 ± 8% control, *p* < 0.001; amplitude: 70 ± 1% of control, *p* = 0.014, Student’s *t*-test, n = 5, Fig. 8b1,d,e). Cumulative distribution plotting of the amplitude and inter-event interval revealed the same conclusion. Specifically, the inter-event interval cumulative distribution of mIPSCs was shifted significantly leftward by 5-HT (*p* = 0.004, K–S test, Fig. 8a2) and α-methylserotonin (*p* = 0.024, K–S test, Fig. 8c2), but rightward by DOI (*p* < 0.001, K–S test, Fig. 8b2); while the amplitude cumulative distribution was substantially shifted rightward only by DOI (DOI, *p* < 0.001; 5-HT, *p* = 0.511; α-methylserotonin, *p* = 0.127; K–S test, Fig. 8a2,b2,c2). Taken together, our data suggested that several subtypes of presynaptic 5-HT_2_ receptor differentially modulated inhibitory synaptic transmission in a manner different from those governing the postsynaptic effect.

## Discussion

Our present study demonstrated presynaptic and postsynaptic 5-HT_2_ receptors functionally regulated inhibitory synaptic transmissions in human iPS-derived neuronal networks, confirming that these induced neurons highly resemble those of naïve forebrain neurons in terms of physiological properties. Our major findings are: (1) Using our present neural induction protocol, NPCs can be stably and efficiently induced from human iPS with no manual selection needed; (2) These iPS-derived NPCs could be differentiated into morphologically and functionally mature cortical neurons; (3) Serotonin modulated GABAa receptors mainly through 5-HT_2A/C_ receptors; (4) Postsynaptically, 5-HT_2A/C_ receptors exerted its modulating effect through GPCR-PLC-PKC, and Ca^2+^ signaling pathway; (5) Presynaptically, the inhibiting effect mediated by 5-HT_2A/C_ receptors was counteracted by other 5-HT_2_ receptors.

Neuronal progenitors generated *in vitro* as donor cells for transplantation hold great promise to treat neurological diseases^6^,^7^,^8^,^11^,^13^,^35^,^36^. Neural stem cells from iPS have advantages over those from ES in term of less ethic concerns and immunity rejections^8^,^12^. Currently virus-free generation of iPS warranted the safety of iPS-derived neurons in translational application^1^,^35^. Increasing evidences indicated that transplanted progenitors could survive, migrate, differentiate, integrate into host neural circuitry, and even correct the behavioral deficits^2^,^3^,^6^,^7^,^13^,^36^. It has already been reported that ES-derived neurons exhibited homeostatic plasticity and BDNF- induced synaptic plasticity^15^, as well as input-specific long-term potentiation^14^. The brain circuitry is known to receive massive monoaminergic projecting fibers engaging in circadian rhythm, motor coordination , learning and memory^37^. The brain function can’t be properly fulfilled without serotonin modulation^16^. GABAergic inhibition balances brain state by opposing glutamatergic excitation^38^,^39^. The crosstalk between GABA and serotonin systems has been justified in the forebrain^23^, where 5-HT_2_ receptors mediate the regulation of GABAergic system both presynaptically and postsynaptically. Therefore the iPS-derived neurons for engrafting should be modulated as its host counterparts, otherwise new disorders might be caused by the transplantation.

Serotonin mainly activated postsynaptic 5-HT_2A/C_ receptors to reduce GABA -activated currents through G protein, PLC, PKC, as well as Ca^2+^ signaling. DOI reduced inhibitory neurotransmitters activated currents in the cortical neurons either through receptor phosphorylation or trafficking^23^,^40^. We postulated that our human iPS-derived neurons likely shared the same mechanism. Presynaptic 5-HT_2_ receptors were also reported to regulate transmitter release^22^,^25^,^41^. Serotonin significantly influenced mIPSCs through 5-HT_2_ receptors in our iPS-derived neural network. The enhancement of mIPSCs by serotonin could be mimicked by 5-HT_2_ receptor agonists. Conversely, a reduction of frequency and amplitude of mIPSCs was caused by DOI. The frequency of mIPSCs is closely related to the probability of transmitter release. The decreased frequency of mIPSCs by DOI means less transmitter release from presynaptic terminals, but this action can be counteracted by other 5-HT_2_ receptors^42^. Overall our findings suggested that 5-HT_2A/C_ receptors not only downregulated postsynaptic GABAa receptors, but also attenuated presynaptic GABA release of the iPS-derived neurons.

Taken together, our findings demonstrate 5-HT_2_ receptors functionally modulated GABAergic synaptic transmission in the neural networks composed of human iPS-derived neurons, suggesting that iPS-derived neurons would receive diffused serotonin neuromodulation after transplantation just like host cells, but also could sever as an ideal *in vitro* model for studying neurological diseases and screening serotonin related neuropsychological drugs.

## Methods

All experiments were carried out in accordance with the guidelines of the Human Subject Research Ethics Committee at Guangzhou Institutes of Biomedicine and Health (GIBH), Chinese Academy of Sciences (CAS), and the Committee approved the experiments. Formal informed consent was obtained from all subjects.

### Cell culture and neural differentiation

Two human iPS lines from healthy human, UC5 (Passage 15–25)^28^ and GZ2 (Passage 10–20)^27^, established and maintained in our laboratory and one human ESCs line, H1 (Passage 40–50, Wicell, Madison, WI, USA), were adopted in the present study. UC5 cell line was derived from urine cells using a feeder-free, serum-free and virus-free method without oncogene c-MYC. GZ2 cell line was reprogrammed from skin fibroblasts by using Yamanaka factors^43^. All these pluripotent cells were cultured as described elsewhere^44^,^45^ on plates coated with Matrigel (BD Biosciences, San Jose, CA, USA) in mTesR1 medium (Stemcell Technologies, Vancouver, BC, Canada), and routinely passaged by EDTA (Ethylene Diamine Tetraacetic Acid, 0.5 mM) dissociation every 4–6 day.

Neural induction was performed as previously reported via a monolayer strategy^46^ by dual inhibition of SMAD signaling with empirical modifications to get highly homogenous neural progenitor cells (NPCs) of dorsal forebrain identity. Briefly, once the pluripotent cells got 100% confluence (Day 0 of neural induction), the medium was changed to neural induction medium (NIM). NIM basically contained N2B27 medium (DMEM/F12: Neurobasal (1:1), 0.5% N2, 1% B27, 1% Glutamax (GIBCO), 1% non-essential amino acid (GIBCO)), plus 2 inhibitors (5 μM S431542 (Sigma), 5 μM Dorsomophin (Sigma)). By the 8-9^th^ day post induction, when a uniform packed neuroepithelial layer appeared routinely, cell aggregates dissociated from the neuroepithelium by manually scratching and gentle pipetting with no selection, were replated on new Matrigel plate, and continually fed with NIM without inhibitors for another 8 days. Then NPCs were harvested by floating the neural rosettes with Dispase digestion without picking and further purified via 1-2 passage(s) with Accutase digestion into single cells. The dorsal forebrain identity of NPCs was confirmed by immunostaining and Q-PCR.

For neuron differentiation, NPCs were digested into single cells with Accutase, plated on Matrigel-coated glass coverslips in 24-well plate at a density of 40,000 cells/well and fed with neural differentiation medium (NDM). NDM consisted of Neurobasal (Life Technologies), 2% B27, 1% Glutamax (GIBCO), and 1% non-essential amino acid (GIBCO), supplemented with BDNF, GDNF, IGF1 (all at 10 ng/ml, Peprotech), ascorbic acid (200 ng/ml) and cAMP (100 nM). Expression of neuron markers Tuj1 and Map2, astrocyte marker GFAP, as well as neuronal subtype markers glutamate and GABA was examined by immunostaining.

### Immunofluoresent microscopy

Immunostaining of cells was done as previously described^1^. Briefly, neural stem cells or differentiated neurons plated on glass coverslips were fixed by 4% PFA solution for 20 min at room temperature, followed by blocking with 5% bovine serum albumin in PBS for 1 h at room temperature (RT). Subsequently, samples were incubated with primary antibodies overnight at 4 °C and then with appropriate fluorescent probe-conjugated secondary antibodies for 1 h at RT. Nuclei were counterstained with DAPI. Images were acquired using Carl Zeiss microscope. The primary antibodies used here included: Sox1 (1:1000, Millipore), Sox2 (1:100, R&D Systems), Nestin (1:200, Millipore), Pax6 (1:300, R&D Systems), Tuj1 (1:500, Covance), Map2 (1:500, Millipore), Glutamate (1:1000, Sigma), GABA (1:1000, Sigma).

### Electrophysiological Recording

Whole-cell patch-clamp recordings were obtained at room temperature from iPS-derived neurons as described previously^26^,^47^. Signals were amplified with a MultiClamp 700B amplifier, digitized with a Digidata 1440, and acquired with pClamp 10 software (Molecular Devices,USA). The bath solution contained (in mM): 127 NaCl, 5 KCl, 2 MgCl_2_, 2 CaCl_2_, 10 HEPES, and 12 glucose (pH 7.4 with NaOH, 300 osmol/L). Patch pipettes with resistance between 8–10 MΩ were pulled from borosilicate glass (WPI, USA) with a Sutter P97 puller (Sutter, USA). Pipettes were filled with solutions containing (in mM): 145 K-gluconate, 0.2 EGTA, 10 HEPES, 5 NaCl, 1 MgCl_2_, 4 Mg-ATP, and 0.3 Na-GTP (pH 7.2 with KOH, 285 osmol/L). In recording GABA activated currents and spontaneous inhibitory synaptic currents, CsCl-based solution was used to get inward chloride currents (in mM):130 CsCl, 4 NaCl, 1 MgCl_2_, 10 HEPES, 5 EGTA, 2 QX-314, 2 MgATP, and 0.2 Na-GTP (pH 7.2 with CsOH, 285 osmol/L).

To directly activate GABAa receptors on the membrane of recorded iPS-derived neuron, GABA (1 mM) was locally applied with a pressure of 15 psi controlled by a custom-made puffing device^48^. An external solution flowed following the GABA delivery to reduce receptor desensitization, and neurons were generally held at −70 mV to record inward ion currents. Gamma-aminobutyric (GABA) receptor-mediated inhibitory postsynaptic currents (IPSCs) were recorded in a voltage-clamp mode. The external solutions including different drugs were exchanged to perfuse the neurons. 6,7-dinitroquinoxaline-2,3-dione (DNQX, 20 μM) and R-2-amino-5- phosphonopentanoate (APV, 50 μM) were used to block glutamatergic transmission. 1 μM TTX was routinely added in the external solution to prevent spontaneous action potentials of the neural networks. Serotonin and α-methylserotonin was purchased from Sigma-Aldrich, and other receptor agonist and antagonists were purchased from Tocris Cookson Ltd.

### Data analysis

Off-line data analysis was performed by using Clampfit 10.2 (Molecular Devices, USA). The Minianalysis (Synaptosoft, USA) was used to extract events for analyzing the frequency and amplitude of spontaneous IPSCs and waveforms of action potentials. Processed data were further imported into Origin 8.0 (OriginLab Corporation, USA) for plotting graphs. Numerical data were reported as mean ± SE (standard error). Student’s *t*-test and Kolmogorov-Smirnov test (K-S test) were used to evaluate significance level unless otherwise stated, and *p* < 0.05 was considered statistically significant.

## Additional Information

**How to cite this article**: Wang, H. *et al*. 5-HT2 receptors mediate functional modulation of GABAa receptors and inhibitory synaptic transmissions in human iPS-derived neurons. *Sci. Rep*. **6**, 20033; doi: 10.1038/srep20033 (2016).
